# Comment to “The beneficial effect of combination therapy with sulfasalazine and valsartan in the treatment of ulcerative colitis [EXCLI Journal 2021;20:236-247]”

**DOI:** 10.17179/excli2025-8388

**Published:** 2025-05-23

**Authors:** Jan G. Hengstler, Agapios Sachinidis

**Affiliations:** 1 Leibniz Research Centre for Working Environment and Human Factors at TU Dortmund (IfADo); 2 Institute of Neurophysiology and Centre for Molecular Medicine Cologne (CMMC), Robert-Koch-Str. 39, 50931 Cologne, Germany

## ⁯⁯⁯

### Editors' inquiry concerning: 

Asgharzadeh F, Yaghoubi A, Nazari SE, Hashemzadeh A, Hasanian SM, Avan A, Javandoost A, Ferns GA, Soleimanpour S and Khazaei M. The beneficial effect of combination therapy with sulfasalazine and valsartan in the treatment of ulcerative colitis. EXCLI J. 2021;20: 236-247. https://doi.org/10.17179/excli2021-3370

We received a notice of possible scientific misconduct concerning the article of Asgharzadeh et al. (2021) published in our journal.

According to our guidelines, we investigate such accusations in a transparent and objective manner. Our investigations include the request and analysis of raw data. However, it is beyond our capabilities to carry out investigations in the laboratories of the authors or to test the reproducibility of the data experimentally ourselves.

In the present case, the approaches were as follows: In Figure 1B of the above mentioned publication (Asgharzadeh et al., 2021), the same or very similar data for controls (negative controls) and colitis (positive controls) were shown also in other publications. Therefore, two members of our editorial board investigated, if the controls of Figure 1B of Asgharzadeh et al. (2021) are adequate. Upon our request, the corresponding author sent us the set of raw data corresponding to Figure 1B. Our conclusion is that the listed negative and positive controls are adequate and agree to the data shown in Figure 1B. It may be criticized that the authors did not mention that the same negative and positive controls were also used for other comparisons published in different articles. However, this does not compromise the conclusions of the here investigated article. In future, the editors will consistently demand that this must be stated in the materials and methods section if data is used more than once, and we will include this rule in our guidelines. 

The table with the raw data of each individual mouse, treatment group, body weight at the different time periods after treatment, and the date of treatment is available in Supplementary Table 1(Tab. 1).

## Figures and Tables

**Table 1:**
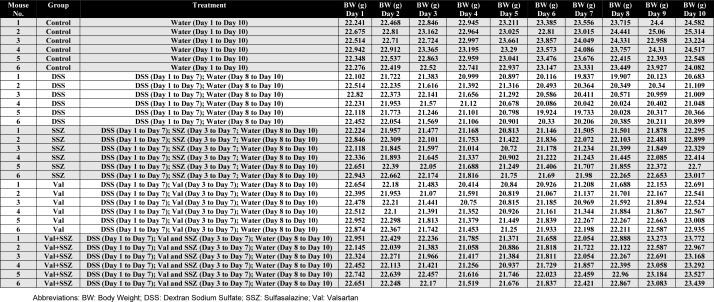
Supplementary Table 1: This table contains the raw data corresponding to Figure 1A and B in the original article: "Asgharzadeh F, Yaghoubi A, Nazari SE, Hashemzadeh A, Hasanian SM, Avan A, Javandoost A, Ferns GA, Soleimanpour S, Khazaei M. The beneficial effect of combination therapy with sulfasalazine and valsartan in the treatment of ulcerative colitis. EXCLI J. 2021;20:236-247. doi: 10.17179/excli2021-3370.

